# Resveratrol Reduces Glucolipid Metabolic Dysfunction and Learning and Memory Impairment in a NAFLD Rat Model: Involvement in Regulating the Imbalance of Nesfatin-1 Abundance and Copine 6 Expression

**DOI:** 10.3389/fendo.2019.00434

**Published:** 2019-07-09

**Authors:** Xing-Xing Chen, Ya-Yun Xu, Rui Wu, Zheng Chen, Ke Fang, Yin-Xiu Han, Yue Yu, Ling-Ling Huang, Lei Peng, Jin-Fang Ge

**Affiliations:** ^1^School of Pharmacy, Anhui Medical University, Hefei, China; ^2^The Key Laboratory of Anti-inflammatory and Immune Medicine, Ministry of Education, Anhui Medical University, Hefei, China; ^3^Department of Pharmacy, The Fourth People's Hospital in Hefei, Hefei, China; ^4^Department of Pharmacy, The People's Hospital of Huangshan, Huangshan, China; ^5^Department of Pharmacy, Lujiang County Hospital of Traditional Chinese Medicine, Hefei, China

**Keywords:** resveratrol, nonalcoholic fatty liver disease (NAFLD), Morris water maze, nesfatin-1, Copine 6, glycogen synthase kinase-3beta (GSK3β)

## Abstract

Resveratrol (RES) is a polyphenolic compound, and our previous results have demonstrated its neuroprotective effect in a series of animal models. The aim of this study was to investigate its potential effect on a nonalcoholic fatty liver disease (NAFLD) rat model. The parameters of liver function and glucose and lipid metabolism were measured. Behavior performance was observed via the open field test (OFT), the sucrose preference test (SPT), the elevated plus maze (EPM), the forced swimming test (FST), and the Morris water maze (MWM). The protein expression levels of Copine 6, p-catenin, catenin, p-glycogen synthase kinase-3beta (GSK3β), GSK3β, and cyclin D1 in the hippocampus and prefrontal cortex (PFC) were detected using Western blotting. The results showed that RES could reverse nesfatin-1-related impairment of liver function and glucolipid metabolism, as indicated by the decreased plasma concentrations of alanine aminotransferase (ALT), aspartate aminotransferase (AST), total bilirubin (TBIL), direct bilirubin (DBIL), indirect bilirubin (IBIL), total cholesterol (TC), low-density lipoprotein cholesterol (LDL-C), glucose, insulin, and nesfatin-1; increase the plasma level of high-density lipoprotein cholesterol (HDL-C); and reduce hepatocyte steatosis in NAFLD rats. Although there was no significant difference among groups with regard to performance in the OFT, EPM, and FST tasks, RES-treated NAFLD rats showed an increased sucrose preference index in the SPT and improved learning and memory ability in the MWM task. Furthermore, the imbalanced protein expression levels of Copine 6, p-catenin, and p-GSK3β in the hippocampus and PFC of NAFLD rats were also restored to normal by treatment with RES. These results suggested that four consecutive weeks of RES treatment not only ameliorated glucolipid metabolic impairment and liver dysfunction in the NAFLD rat model but also mitigated the attendant behavioral and cognitive impairments. In addition to the mediating role of nesfatin-1, the mechanism underlying the therapeutic effect of RES on NAFLD might be associated with its ability to regulate the imbalanced expression level of Copine 6 and the Wnt signaling pathway in the hippocampus and PFC.

## Introduction

Nonalcoholic fatty liver disease (NAFLD) is a clinicopathological syndrome characterized by hepatic steatosis without significant alcohol use or other known liver disease. With an estimated prevalence of 25% in the general population (1, 2), NAFLD is considered the most common cause of chronic liver disease (3, 4). Moreover, the clinical burden of NAFLD is not restricted to liver-related morbidity or mortality (5), fueling concerns about the extrahepatic diseases accompanied with or induced by NAFLD. NAFLD is strongly associated with obesity, type 2 diabetes mellitus (T2DM), dyslipidemia, and hypertension and is now regarded as a liver manifestation of metabolic syndrome (6), and increasing evidence has demonstrated a close association between NAFLD and neuropsychiatric diseases including depression and cognitive dysfunction (7). Consistently, in our previous study (8), rats with NAFLD induced by a high-fat diet showed not only metabolic dysfunction, including obesity, liver dysfunction, dyslipidemia, and glucose metabolic dysfunction, but also significant impairment of learning and memory. Many measures have been taken directly toward improving the metabolic status of the liver as well as cell stress, inflammation, and fibrosis (4). However, there are currently no approved therapies for NAFLD, and standard-of-care lifestyle advice is rarely effective (1, 5). Thus, it is imperative to investigate the pathogenesis of NAFLD and explore potential therapeutic targets.

Although the mechanisms underlying the pathogenesis and progression of NAFLD are still incompletely understood, insulin resistance and dyslipidemia related to metabolic syndrome are believed to be the main pathogenic trigger that precipitates the development of NAFLD. It has been reported that insulin resistance is the linkage between NAFLD and T2DM (6), and NAFLD has been considered the hepatic manifestation of insulin resistance (9). Moreover, the homeostasis model assessment parameter of insulin resistance is considered an independent predictive factor for advanced fibrosis in nondiabetic patients with NAFLD, and modulation of insulin resistance is a potential strategy for NAFLD treatment (1). Increased hepatic expression of dipeptidyl peptidase 4 (DPP4) and high serum DPP4 activity have been demonstrated to be associated with NAFLD (10, 11), and NAFLD has been reported to be an independent predictor of the effect of sitagliptin (STG), an oral DPP4 inhibitor, in patients with T2DM (12). Liver-specific DPP4 transgenic mice presented not only elevated systemic DPP4 activity but also a NAFLD-associated syndrome including obesity, hypercholesterolemia, hepatic steatosis, and liver damage (11). Furthermore, these dysfunctions were accompanied by increased expression of peroxisome proliferator-activated receptor gamma (PPARγ) and severe insulin resistance in the liver (11). The efficacy of STG has been demonstrated in NAFLD patients with T2DM, with significant decreases in plasma glucose and serum hemoglobin A1c (HbA1c), aspartate aminotransferase (AST), and alanine aminotransferase (ALT) levels (13). Similarly, as PPARγ ligands, thiazolidinedione antidiabetic agents have been extensively evaluated in the treatment of NAFLD (14, 15). More importantly, in line with the results from animal studies showing that STG has neuroprotective activity including antinociceptive, antidepressant, and cognitive improvement (16), it has also been demonstrated in human research that in addition to the effects of glycemic control, STG therapy may result in the improvement of cognitive function in elderly diabetic patients with and without Alzheimer's disease (AD) (17).

Resveratrol (trans-3,5,4′-trihydroxy-trans-stilbene; RES) is a polyphenol component with diverse beneficial biological and pharmacological activity. It has been reported that RES is capable of ameliorating insulin resistance and improving insulin sensitivity (18). Focusing on its neuroprotective effect, the results of our previous studies have demonstrated that RES can bind to and interfere with the abnormal aggregation of amyloid beta (19), alleviate the impairment of learning and memory (20), and exert an antidepressant-like effect on rat models of chronic unpredictable mild stress (21), and subclinical hypothyroidism (SCH) (22). Based on these findings, it is rational to hypothesize that RES might be a good strategy for therapeutic intervention in NAFLD, targeting not only the metabolic dysfunction but also the behavioral and cognitive impairments. Thus, the main aim of the present study was to testify the effect of RES on NAFLD and explore the possible mechanism.

The novel satiety factor nesfatin-1 and its precursor nucleobindin-2 (NUCB2) were first reported to regulate appetite and food intake (23). Subsequently, increasing evidence has demonstrated the role of nesfatin-1 in regulating not only glucose and energy metabolism (24) but also mood and cognitive function (25, 26).

Copines are a conserved cytosolic protein family characterized by two C2 domains with the ability to bind phospholipids in a calcium-dependent manner (27). Copine 6 is a member of the copine family. It has been reported that Copine 6 is expressed in the postnatal brain, with peak expression in the hippocampus, and is necessary for brain-derived neurotrophic factor (BDNF) to increase the abundance of mushroom spines on hippocampal neurons (27, 28). Copine 6 has been demonstrated to link activity-triggered calcium signals to spine structural plasticity necessary for learning and memory (27) and to regulate BDNF-dependent changes in dendritic spine morphology to promote synaptic plasticity (28). The results of our previous study also showed that BDNF-related imbalance in the expression of Copine 6 and synaptic plasticity markers in both the hippocampus and the prefrontal cortex (PFC) was coupled with depression-like behavior and immune activation in a stressed rat model (29). The Wnt/β-catenin signaling pathway regulates many crucial pathophysiological processes, including not only hepatic homeostasis and liver function (30) but also cognition and mood regulation (31). Dysregulation of glycogen synthase kinase-3beta (GSK3β) activity has been reported in insulin resistance, T2DM, and neurodegenerative diseases (32). Consistently, our previous research has also shown that metabolic disorders and impaired learning and memory in NAFLD rats are involved in the imbalance of nesfatin-1 abundance and Copine 6 expression, as well as the imbalance of the Wnt/β-catenin signaling pathway (8). However, it is unknown whether these changes can be reversed by RES.

To investigate the potential effect of RES on NAFLD and explore the possible mechanism, a NAFLD rat model was established using high-fat diet in the present study, and the protein expression levels of p-GSK3β/GSK3β, p-catenin/catenin, cyclin D, and Copine 6 in the hippocampus and PFC were measured using Western blotting. Blood glucose and lipid concentrations and liver function were measured, and STG and rosiglitazone (RSG) were used as positive control agents. Behavioral performance was examined using the open field test (OFT), the sucrose preference test (SPT), the elevated plus maze (EPM) test, the forced swimming test (FST), and the Morris water maze (MWM); the plasma concentrations of nesfatin-1, leptin, and insulin were also measured. Fluoxetine (FLX) and donepezil (DNP), which are typical drugs used clinically against depression and cognitive impairment, respectively, were used as the positive control agents in the behavioral tests in the present study.

## Materials and Methods

### Drugs

RES was purchased from Sigma Chemical Co. Sitagliptin phosphate (Januvia) was produced by Merck Sharp Dohme Ltd. Rosiglitazone was produced by Chengdu Hengrui Pharmaceutical Co., Ltd. Fluoxetine hydrochloride (Prozac) was provided by Eli Lilly Pharmaceuticals. Donepezil hydrochloride (Haosen) was produced by Jiangsu Haosen Pharmaceutical Co., Ltd. All the drugs were dissolved in an aqueous solution of 0.5% sodium carboxymethyl cellulose for a mixed suspension.

### Animals and Groups

**Figure 1:**
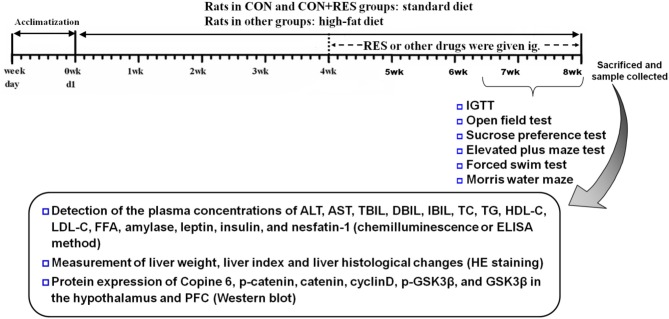
The outline of the experimental design.

Male Sprague–Dawley (SD) rats aged 2 months were randomly divided into eight groups, namely, control (CON) group, CON + RES (15 mg/kg) group, NAFLD group, NAFLD + RES (15 mg/kg) group, NAFLD + STG (10 mg/kg) group, NAFLD + RSG (5 mg/kg) group, NAFLD + FLX (2 mg/kg) group, and NAFLD + DNP (1 mg/kg) group, with five rats in the CON + RES group and eight rats in each of the other groups. The rats were housed with four to five animals per cage (43 cm long ×31 cm wide ×19 cm high) and maintained under a 12:12-h light/dark cycle (lights on at 0700 h) at an ambient temperature of 21–22°C and 50–60% relative humidity. The diets fed to the rats were as described in our previous study (8). Briefly, the rats in the CON and CON + RES groups were administered a standard diet (3,601 kcal/kg; 10% fat, 75.9% carbohydrates, and 14.1% protein as percent of kcal; Trophic Animal Feed High-Tech Co., Ltd., China). Rats in other groups were given a high-fat diet (5,000 kcal/kg; 60% fat, 25.9% carbohydrate, and 14.1% protein as percent of kcal) supplied by the same company. Starting 4 weeks later, RES and the other drugs were administered intragastrically for 4 weeks. The CON and untreated NAFLD model rats received daily intragastric injections of 0.5% sodium carboxymethyl cellulose. The outline of the experimental design is shown in Figure 1.

All experimental procedures in the present study were approved by the Animal Care and Use Committee of Anhui Medical University in compliance with the National Institutes of Health (NIH) *Guide for the Care and Use of Laboratory Animals* (NIH publication no. 85-23, revised 1985).

### Behavioral Tests

All behavioral tests were performed according to our previous studies (8, 22). They were conducted during the light phase of the light/dark cycle in a separate room similar to the housing room, and the timing of the tests was matched between groups. The animals were allowed to adapt to the testing environment for 20 min before each test, and the observers were blinded to the treatment. The performance of the rats was monitored and recorded using a digital camera interfaced with a computer with ANY-maze video imaging software (Stoelting Co., Wood Dale, USA).

The behavioral tests began with the OFT, followed by the SPT, EPM, FST, and MWM in that order.

### Open Field Test

The apparatus consisted of a black 100-cm ×100-cm square arena with a 30-cm black high wall. The floor was marked with a grid dividing the floor into 16 equal-sized squares. During the 5-min observation period, the rats were placed in one corner of the apparatus, facing the wall. The total distance, the distance moved in the center, and the frequencies of rearing and grooming were recorded.

### Sucrose Preference Test

After a 12-h period of food and water deprivation, all rats were provided free access to two bottles containing plain water or 2% sucrose solution. After 6 h, the volumes of water and sucrose solution consumed by the rats were measured. The sucrose preference index (SPI), which is the percentage of sucrose solution out of the total volume of liquid ingested, was used as a measure for anhedonia.

### Elevated Plus Maze Test

The maze (made of Plexiglas) consisted of a plus-shaped apparatus, with two opposite closed arms (45 cm ×11 cm) enclosed by walls (22 cm in height) and two opposite open arms (45 cm ×11 cm) without walls. The apparatus also had a central arena (11 cm ×11 cm) and was elevated 80 cm above the floor. Each rat was placed in the central arena of the maze facing an open arm and allowed to explore the maze for 5 min. The distances moved in the open arms and the closed arms were analyzed.

### Forced Swimming Test

The cylinder for this behavioral test was 60 cm tall and 25 cm in diameter maintained at 24–25°C and filled with 30 cm of water. The FST paradigm includes two sections: an initial 15-min pretest followed by a 5-min test administered 24 h later. The immobility time was recorded, and the rats were considered immobile when they did not make any active movements.

### Morris Water Maze Test

A pool (1.8 m in diameter) was filled with opaque water and surrounded by complex maze cues. An escape platform (9 cm in diameter) was placed in the center of a designated quadrant with its top 2 cm below the water surface. In the hidden-platform acquisition phase, each rat performed four training trials per day for 4 days. In each trial in the hidden-platform test, the rat was given 60 s to find the platform and allowed to stay there for 20 s. If a rat failed to find the hidden platform within 60 s, then it was guided to the platform and allowed to remain there for 20 s. A probe test was conducted on day 5, during which the hidden platform was removed from the pool and the rat was allowed to swim for 60 s. The escape latency (latency to find the platform) in the acquisition phase and the duration spent in the target quadrant in the probe test were analyzed.

### Intraperitoneal Glucose Tolerance Test

An intraperitoneal glucose tolerance test (IGTT) was conducted in rats after 12 h of fasting and water deprivation. Glucose was injected intraperitoneally at a dose of 2.0 g/kg, and whole blood was collected from the tail tip before injection and 15, 30, 60, and 120 min after injection. Blood glucose was measured using a Roche glycemic meter.

### Measurement of the Plasma Concentrations of ALT, AST, Total Bilirubin, Direct Bilirubin, Indirect Bilirubin, Total Cholesterol, Triglyceride, High-Density Lipoprotein Cholesterol, Low-Density Lipoprotein Cholesterol, Free Fatty Acids, Amylase, Leptin, Insulin, and Nesfatin-1

Twenty-four hours after the last behavioral test, the rats were deeply anesthetized with chloral hydrate, and blood was drawn from the abdominal aorta. The plasma concentrations of ALT, AST, total bilirubin (TBIL), direct bilirubin (DBIL), indirect bilirubin (IBIL), total cholesterol (TC), triglyceride (TG), high-density lipoprotein cholesterol (HDL-C), low-density lipoprotein cholesterol (LDL-C), free fatty acids (FFAs), and amylase were detected using chemiluminescence. The plasma levels of leptin, insulin, and nesfatin-1 were measured using commercially available enzyme-linked immunosorbent assay (ELISA) kits (leptin and insulin: Yuanye Biotech. Co., LTD, Shanghai, China; nesfatin-1: Cusabio Biotech. Co., LTD, Wuhan, China) according to the manufacturers' instructions.

### Measurement of Liver Weight, Liver Index, and Liver Histological Changes

After the blood was collected, the liver was rapidly dissected and weighted, and the liver index (liver weight/100 g bodyweight) was calculated. Four rats in each group were randomly selected, and the same part of the liver was collected, fixed with 1% neutral-buffered formalin, embedded in paraffin, sectioned at a thickness of 4 μm, and stained with hematoxylin and eosin (HE). The changes in the liver were scored according to the method used in our previous study (8). Briefly, the liver was graded according to gross appearance (0 = no changes in color or consistency; 1 = pale and yellowing; 2 = mottled, pale, and yellowing; 3 = mottled, pale, and yellowing and smooth rounded edges). The extent of fatty change in the liver was graded according to the amount and size of lipid-filled vacuoles presented throughout the stained sections (0 = no evidence of lipid vacuoles; 1 = few small lipid vacuoles present within hepatocytes; 2 = increased number and larger lipid vacuoles within hepatocytes).

### Western Blot Assays

The hippocampi and the PFCs from three rats in each group were rapidly dissected, frozen in liquid nitrogen, and stored at −80°C. The tissues were homogenized in radioimmunoprecipitation assay (RIPA) buffer (50 mM tris-HCl, pH 7.4, 0.1% SDS, 1% NP-40, 0.25% sodium deoxycholate, 150 mM NaCl, 1 mM EDTA, 1 mM EGTA, and 1 mM Na_3_VO_4_). Before homogenization, protease inhibitor cocktail (Roche, Indianapolis, USA) and the phosphatase inhibitor PhosSTOP (Roche, Indianapolis, USA) were added. Protein quantitation was conducted using a Lowry Protein Assay Kit (Meiji Biotech. Co., Ltd., Shanghai, China). The same quantity (50 μg) of protein from each animal was loaded and separated using 15% sodium dodecyl sulfate polyacrylamide gel electrophoresis (SDS-PAGE) and subsequently transferred onto a polyvinylidene difluoride membrane (Amersham Biosciences, UK). The membrane was blocked with 5% skim milk for 1 h and then incubated with antibodies targeting Copine 6 (1:1,000; Santa Cruz Biotechnology, Inc)., β-catenin (1:800; Cell Signaling Technology), p-β-catenin (1:800; Cell Signaling Technology), cyclin D1 (1:1,000; Abcam Technology), GSK3β (1:1,000; Cell Signaling Technology), p-GSK3β (1:800; Cell Signaling Technology), or β-actin (1:1,000; Zhongshan Biotechnology, Inc., Beijing, China) at 4°C overnight and subsequently incubated with a horseradish peroxidase-conjugated secondary antibody (1:2,000) at 37°C for 2 h. The blots were developed using the Easy Enhanced Chemiluminescence Western Blot Kit (Pierce Biotechnology, Rockford, IL, USA). Protein bands were scanned and analyzed using the ImageJ software (NIH).

### Statistical Analysis

All statistical analyses were performed using SPSS (Statistical Package for the Social Sciences) version 12.0.1 (SPSS Inc., Chicago, IL, USA). The data are expressed as the mean ± standard error of the mean (SEM), and *P* < 0.05 was considered statistically significant.

The distribution of the data was determined by the Kolmogorov–Smirnov test. Between-group effects on bodyweight and escape latency in the MWM task were analyzed by repeated-measures analysis of variance (ANOVA) with group and time as the factors followed by the least significant difference (LSD) *post hoc* test. Statistical analyses of other parameters were carried out using ANOVA followed by the LSD *post hoc* test. Correlation analysis was performed using a Pearson's correlation test.

## Results

### RES Administration Alleviated Hepatomegaly, Hepatocyte Steatosis, and Liver Dysfunction in NAFLD Rats

**Figure 2:**
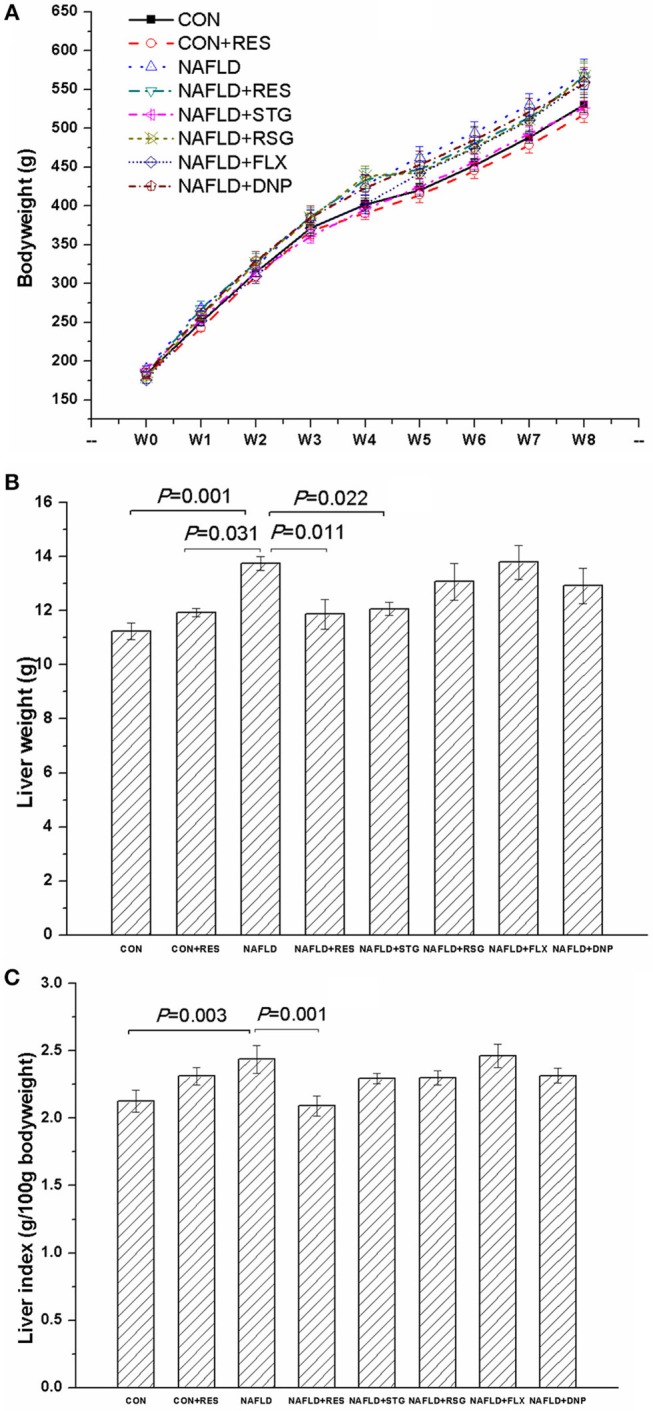
Effect of RES on the bodyweight, liver weight, and liver index of NAFLD rats. The data are presented as the mean ± SEM, with five rats in the CON + RES group and eight rats in each of the other groups. The bodyweight is shown in panel **(A)**, and results of the repeated-measures ANOVA showed a significant effect of time but not treatment on the bodyweight, with a significant interaction between treatment and week. The NAFLD model rats showed greater liver weight **(B)** and a higher liver index **(C)** than the control or CON + RES rats. Treatment with RES reversed the increase in liver weight and liver index but had no significant effect on bodyweight.

Figure 2 shows the changes in bodyweight, liver weight, and liver index in all the groups. In an analysis of the bodyweight using repeated-measures ANOVA with experimental treatment as a between-subject factor and week as a within-subject factor, there was a significant effect of time on the bodyweight [*F*_(8, 432)_ = 3,008.986, *P* < 0.01], with a significant interaction between treatment and week [*F*_(56, 432)_ = 1.848, *P* < 0.01]. However, the factor of treatment did not affect bodyweight significantly [*F*_(7, 53)_ = 1.207, *P* = 0.315].

As shown in Figures 2B,C, the liver weight and liver index were increased in the NAFLD group compared with the CON group (*P* < 0.05 or *P* < 0.01). Treatment with STG reversed the increase in liver weight (*P* = 0.022) but not the increase in liver index, whereas RES reversed the increase in both variables (*P* < 0.05 or *P* < 0.01).

**Figure 3:**
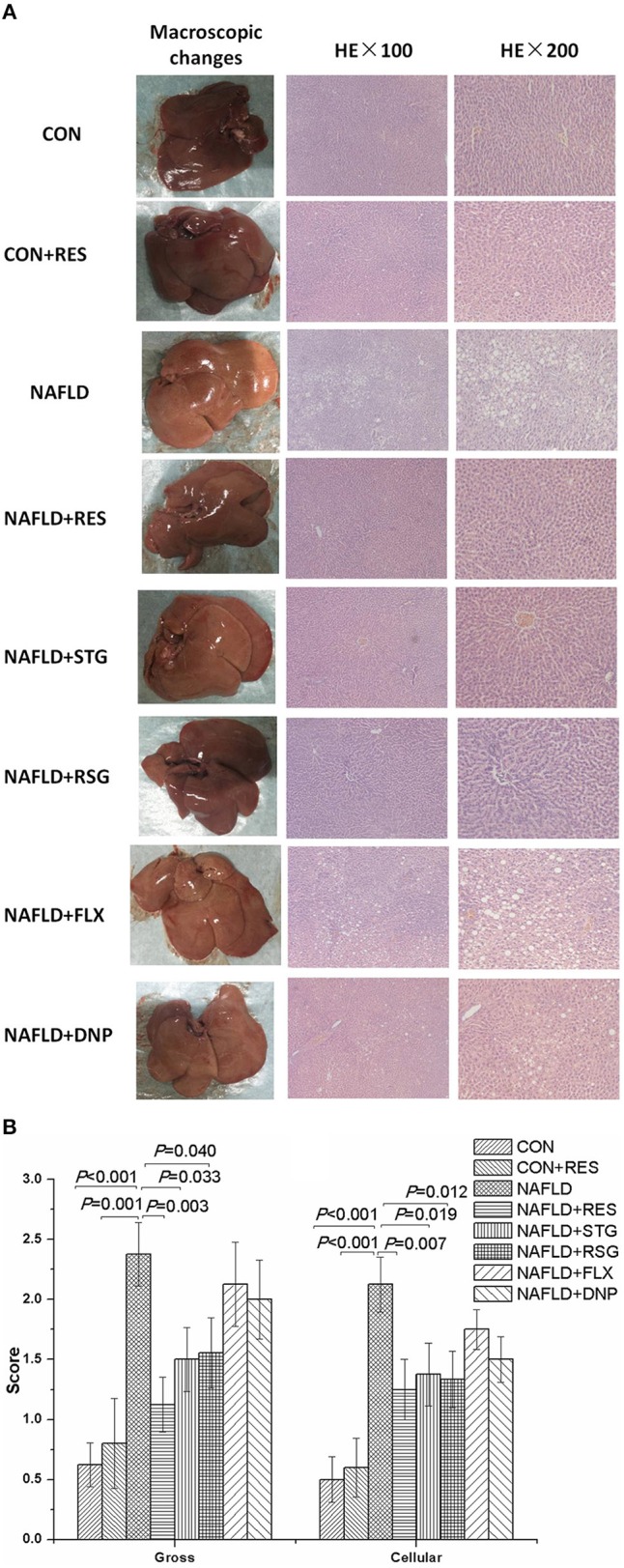
Effect of RES on the macroscopic and histological liver changes in NAFLD rats. The livers of the NAFLD rats appeared yellow or pale, greasy, and brittle. Inflammatory cells and numerous lipid droplets were detected in the livers of NAFLD rats via HE staining **(A)**. The gross and cellular scores of the liver histological changes in the NAFLD model rats were significantly increased **(B)**. Compared with those in the NAFLD group, the color and texture of the livers of the RES-treated rats were closer to normal, and the gross and cellular scores were significantly decreased.

Figure 3 shows the macroscopic and microscopic histological changes in the livers in the different groups. No macroscopic change was observed in the livers of the rats in the CON group, but the livers of the NAFLD rats appeared yellow or pale, greasy, and brittle. The results of the HE staining revealed inflammatory cells and numerous lipid droplets in the livers of NAFLD rats, indicating inflammation and mild hepatocyte steatosis in the liver. However, the livers of the NAFLD + RES group showed less inflammation and hepatocyte steatosis than those of the NAFLD group. As shown in Figure 3B, the gross and cellular injuries of the livers were improved in the RES-treated group (*P* < 0.01).

As shown in Table 1, the plasma concentrations of ALT, AST, TBIL, DBIL, and IBIL in the NAFLD group were all significantly higher than those in the CON group (*P* < 0.01). Treatment with RES, STG, or RSG reversed these changes (*P* < 0.05 or *P* < 0.01).

**Table 1:** Effect of RES on the plasma concentrations of ALT, AST, TBIL, DBIL, and IBIL in NAFLD rats.

**Group**	**Dose (mg/kg)**	***n***	**ALT (U/L)**	**AST (U/L)**	**TBIL (μM)**	**DBIL (μM)**	**IBIL (μM)**
CON	–	8	33.50 ± 2.34	141.38 ± 3.72	3.55 ± 0.14	0.78 ± 0.06	2.67 ± 0.16
CON + RES	15	5	32.02 ± 1.70*^##^*	136.60 ± 1.75*^##^*	3.10 ± 0.08*^##^*	0.80 ± 0.04*^##^*	2.30 ± 0.11*^##^*
NAFLD	–	8	67.45 ± 2.28^**^	171.62 ± 3.20^**^	6.34 ± 0.18^**^	2.36 ± 0.38^**^	3.96 ± 0.22^**^
NAFLD + RES	15	8	36.66 ± 1.14*^##^*	143.38 ± 2.09*^##^*	4.00 ± 0.17*^##^*	1.25 ± 0.14*^##^*	2.88 ± 0.13*^##^*
NAFLD + STG	10	8	32.74 ± 1.36*^##^*	142.25 ± 3.01*^##^*	3.72 ± 0.08*^##^*	0.82 ± 0.04*^##^*	2.90 ± 0.12*^##^*
NAFLD + RSG	5	8	29.79 ± 1.82*^##^*	138.33 ± 3.42*^##^*	3.80 ± 0.10*^##^*	0.87 ± 0.06*^##^*	2.93 ± 0.10*^##^*
NAFLD + FLX	2	8	62.02 ± 1.91^#^	168.88 ± 2.20	5.48 ± 0.24*^##^*	1.62 ± 0.25*^##^*	3.79 ± 0.18
NAFLD + DNP	1	8	62.19 ± 2.03	167.88 ± 3.06	5.34 ± 0.18*^##^*	1.59 ± 0.13*^##^*	3.78 ± 0.14

** P < 0.001 compared with the CON group;

# P < 0.05 and

## *P < 0.001 compared with the NAFLD model group*.

### RES Administration Alleviated the Glucose and Lipid Metabolic Disorder in NAFLD Rats

**Figure 4:**
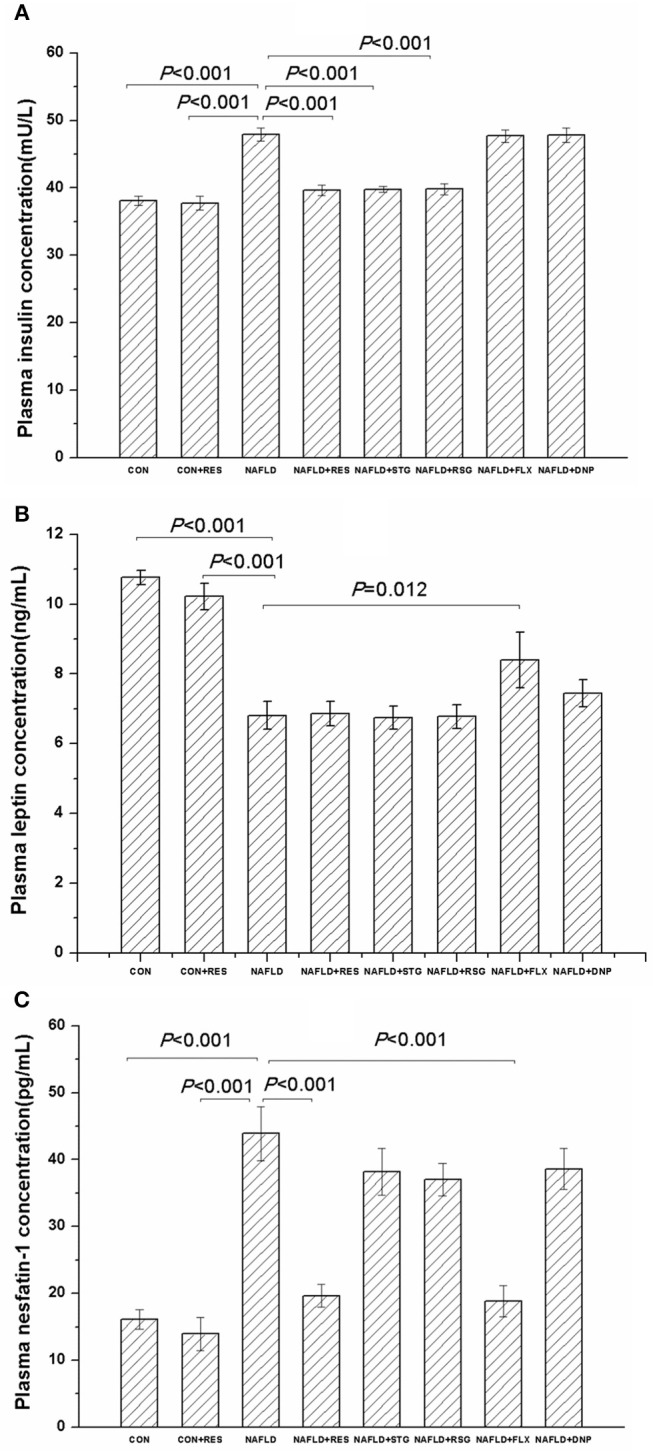
Effect of RES on the plasma concentrations of insulin, leptin, and nesfatin-1 in NAFLD rats. The data are presented as the mean ± SEM, with five rats in the CON + RES group and eight rats in each of the other groups. Compared with the control and CON + RES rats, the NAFLD group showed increased plasma insulin **(A)** and nesfatin-1 **(C)** levels, while the plasma leptin **(B)** level was decreased. Treatment with RES decreased the plasma concentrations of insulin and nesfatin-1 but had no effect on the plasma leptin level.

Compared with those in the control and CON + RES rats, the plasma concentrations of TC, TG, and LDL-C were significantly increased in the NAFLD model group, while the plasma HDL-C level was decreased, and there was no significant difference among groups with regard to the plasma concentration of very low-density lipoprotein-cholesterol (VLDL-C), FFA, or amylase (Table 2). As shown in Table 3 and Figure 4, the blood glucose concentrations 0, 15, 30, and 60 min after injection of glucose were all increased in the NAFLD model group (*P* < 0.05 or *P* < 0.01), as were the plasma insulin and nesfatin-1 levels (*P* < 0.01), while the plasma leptin levels were decreased (*P* < 0.01). Treatment with RES reversed the impaired glucose tolerance and increased plasma insulin and nesfatin-1 levels of NAFLD rats (*P* < 0.05 or *P* < 0.01) but had no effect on the plasma leptin level.

**Table 2:** Effect of RES on the plasma concentrations of TC, TG, HDL-C, LDL-C, VLDL-C, FFA, and amylase in NAFLD rats.

**Group**	**Dose (mg/kg)**	***n***	**TC (mM)**	**TG (mM)**	**HDL-C (mM)**	**LDL-C (mM)**	**VLDL-C (mM)**	**FFA (mM)**	**Amylase (U/L)**
CON	–	8	1.91 ± 0.04	0.65 ± 0.03	2.21 ± 0.05	0.46 ± 0.02	11.71 ± 0.66	926.44 ± 28.47	2,307.12 ± 128.36
CON + RES	15	5	1.94 ± 0.02^*##*^	0.64 ± 0.02^#^	2.12 ± 0.06^*##*^	0.43 ± 0.02^*##*^	10.20 ± 0.52	947.80 ± 24.38	2,345.00 ± 118.06
NAFLD	–	8	2.51 ± 0.05^**^	0.74 ± 0.03^*^	1.76 ± 0.02^**^	0.78 ± 0.02^**^	10.52 ± 0.26	893.57 ± 15.62	2,210.25 ± 101.61
NAFLD + RES	15	8	1.90 ± 0.04^*##*^	0.69 ± 0.02	2.01 ± 0.04^*##*^	0.54 ± 0.02^*##*^	12.10 ± 0.40	933.53 ± 22.59	2,238.62 ± 72.91
NAFLD + STG	10	8	1.98 ± 0.04^*##*^	0.66 ± 0.02	2.01 ± 00.03^*##*^	0.52 ± 0.02^*##*^	13.53 ± 1.04	913.97 ± 20.18	2,255.25 ± 62.34
NAFLD + RSG	5	8	2.01 ± 0.02^*##*^	0.65 ± 0.03^#^	2.00 ± 0.03^*##*^	0.51 ± 0.03^*##*^	10.19 ± 0.40	877.79 ± 12.07	2,265.44 ± 66.23
NAFLD + FLX	2	8	2.51 ± 0.05	0.75 ± 0.04	1.74 ± 0.03	0.74 ± 0.03	13.51 ± 0.87	907.73 ± 16.74	2,102.12 ± 64.48
NAFLD + DNP	1	8	2.44 ± 0.06	0.66 ± 0.04	1.82 ± 0.04	0.78 ± 0.02	10.94 ± 0.64	921.20 ± 20.54	2,038.12 ± 140.68

* P < 0.05 and

** P < 0.001 compared with the CON group;

# P < 0.05 and

## *P < 0.001 compared with the NAFLD model group*.

**Table 3:** Effect of RES on the blood glucose concentration of NAFLD rats in the IGTT.

**Group**	**Dose (mg/kg)**	***n***	**Blood glucose concentration (mM)**				
			**0 min**	**15 min**	**30 min**	**60 min**	**120 min**
CON	–	8	4.48 ± 0.09	15.77 ± 0.78	15.50 ± 1.01	12.40 ± 0.97	9.05 ± 0.47
CON + RES	15	5	4.58 ± 0.11^*##*^	13.2 ± 1.44^*##*^	12.64 ± 1.45^*##*^	11.18 ± 1.92^#^	8.14 ± 0.91
NAFLD	–	8	7.10 ± 0.16^**^	25.40 ± 1.40^**^	22.22 ± 1.36^**^	14.14 ± 1.45	9.23 ± 0.75
NAFLD + RES	15	8	4.56 ± 0.09^*##*^	16.32 ± 0.56^*##*^	13.81 ± 0.64^*##*^	10.81 ± 0.34^#^	8.31 ± 0.15
NAFLD + STG	10	8	4.72 ± 0.13^*##*^	15.46 ± 0.67^*##*^	13.98 ± 0.82^*##*^	11.06 ± 0.59^#^	8.25 ± 0.33
NAFLD + RSG	5	8	4.67 ± 0.10^*##*^	15.56 ± 0.64^*##*^	12.80 ± 0.48^*##*^	10.70 ± 0.37^#^	8.49 ± 0.14
NAFLD + FLX	2	8	7.05 ± 0.22	23.64 ± 1.57	21.32 ± 1.22	12.31 ± 0.79	8.56 ± 0.44
NAFLD + DNP	1	8	6.78 ± 0.16	22.32 ± 0.76^#^	20.70 ± 1.21	13.09 ± 1.11	8.36 ± 0.46

** P < 0.001 compared with the CON group;

# P < 0.05 and

## *P < 0.001 compared with the NAFLD model group*.

The results of Pearson's correlation analysis showed that the plasma nesfatin-1 concentration was positively correlated with the plasma concentrations of TC (*r* = 0.309, *P* = 0.015), LDL-C (*r* = 0.378, *P* = 0.003), and insulin (*r* = 0.318, *P* = 0.013), but negatively correlated with the plasma concentration of HDL-C (*r* = −0.339, *P* = 0.008) and leptin (*r* = −0.531, *P* < 0.001).

### RES Administration Mitigated the Decrease in the SPI of NAFLD Rats in the SPT Without Significant Changes Among Groups in the OFT, EPM, or FST

**Figure 5:**
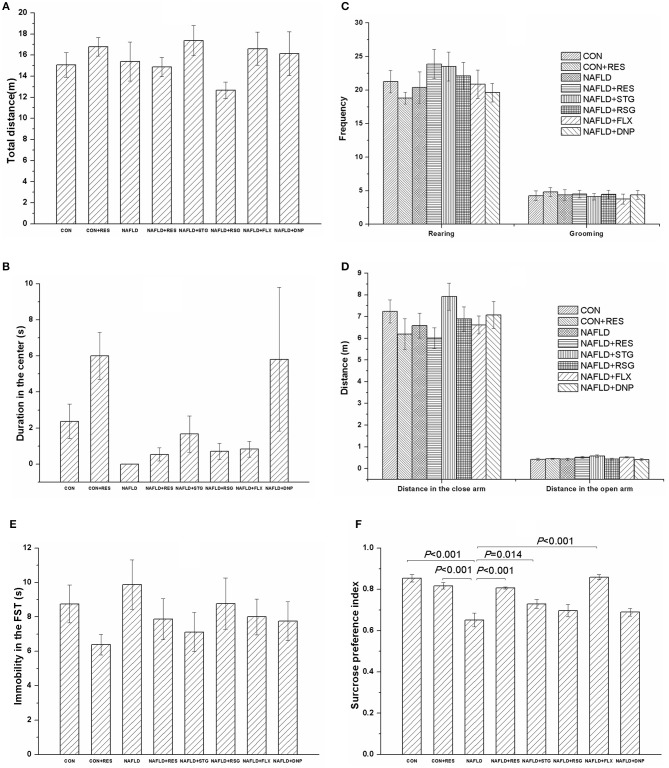
Effect of RES on the behavioral performance of NAFLD rats in the OFT, EPM, FST, and SPT. The data are presented as the mean ± SEM, with five rats in the CON + RES group and eight rats in each of the other groups. In the OFT, there was no significant difference among groups with respect to the total distance traveled **(A)**, the distance traveled in the center **(B)**, or the frequency of rearing or grooming **(C)**. There was no change in performance in the EPM **(D)** or FST **(E)** among the groups. Compared with those of the control and CON + RES groups, the SPI of the rats in NAFLD model group was reduced **(F)**, and this reduction could be reversed by treatment with RES, STG, or FLX **(F)**.

Figure 5 shows the performance of rats in the behavioral tests of the locomotor activity; anxiety, exploration, and despair behaviors; and anhedonia. In the OFT, there was no significant difference in the total distance traveled (Figure 5A), the distance traveled in the center of the field (Figure 5B), or the frequency of rearing or grooming (Figure 5C) among groups.

There was no significant difference in the EPM (Figure 5D) or FST (Figure 5E) performance among the groups. Compared with those of the control and CON + RES groups, the SPI of rats in the NAFLD group was reduced (*P* < 0.01, Figure 5F), and treatment with RES, STG, and FLX mitigated the decrease in the SPI of the NAFLD rats (*P* < 0.05 or *P* < 0.01, Figure 5F).

The results of the Pearson's correlation analysis showed that the plasma nesfatin-1 concentration was negatively correlated with the SPI (*r* = −0.607, *P* < 0.001).

### RES Administration Improved the Impaired Learning and Memory Ability of NAFLD Rats in the MWM Task

**Figure 6:**
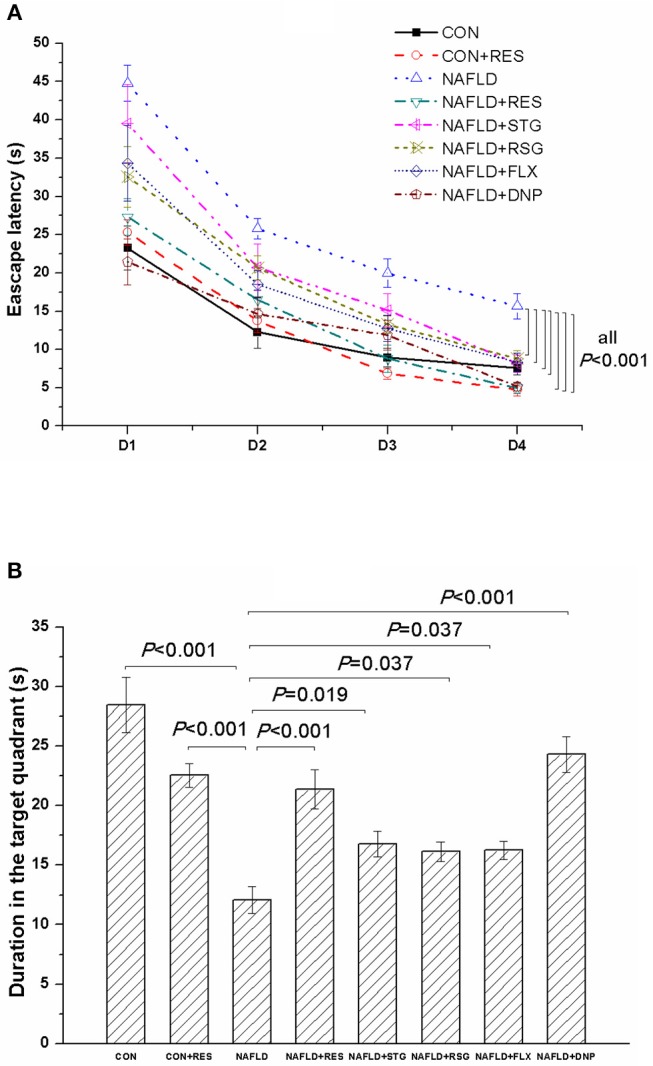
Effect of RES on the behavioral performance of NAFLD rats in the MWM. The data are presented as the mean ± SEM, with five rats in the CON + RES group and eight rats in each of the other groups. In the acquisition phase, the escape latency of the NAFLD rats was longer than that of the control or CON + RES rats on all 4 days **(A)**. In the probe trial, NAFLD rats spent less time than control rats in the target quadrant **(B)**. These abnormalities could be reversed by treatment with RES.

For all the rats studied in this experiment, the escape latency declined with each day during the acquisition phase, as shown in Figure 6A. The results of the repeated-measures ANOVA revealed that both training day [*F*_(3, 159)_ = 148.388, *P* < 0.01] and experimental treatment [*F*_(7, 53)_ = 16.667, *P* < 0.01] had significant effects on the escape latency but did not have a significant interaction effect [*F*_(21, 159)_ = 1.433, *P* = 0. 110]. Results of the LSD *post hoc* test showed that NAFLD rats took longer time to find the submerged platform than did the control (*P* < 0.01) or CON + RES (*P* < 0.01) rats on all 4 days, and the RES-treated rats also had a shorter escape latency than the NAFLD rats did (*P* < 0.01).

In the probe test (Figure 6B), compared with those of the control and CON + RES groups, the duration that the NAFLD rats spent in the target quadrant was decreased (*P* < 0.01). However, the RES-treated rats spend more time in that quadrant than the NAFLD rats did (*P* < 0.01). Notably, a negative correlation was observed between the duration spent in the target quadrant and the plasma concentration of nesfatin-1 (*r* = −0.338, *P* = 0.008).

### RES Administration Imbalanced the Protein Expression Levels of Copine 6 and the Canonical Wnt Pathway in the Hippocampus and PFC of NAFLD Rats

**Figure 7:**
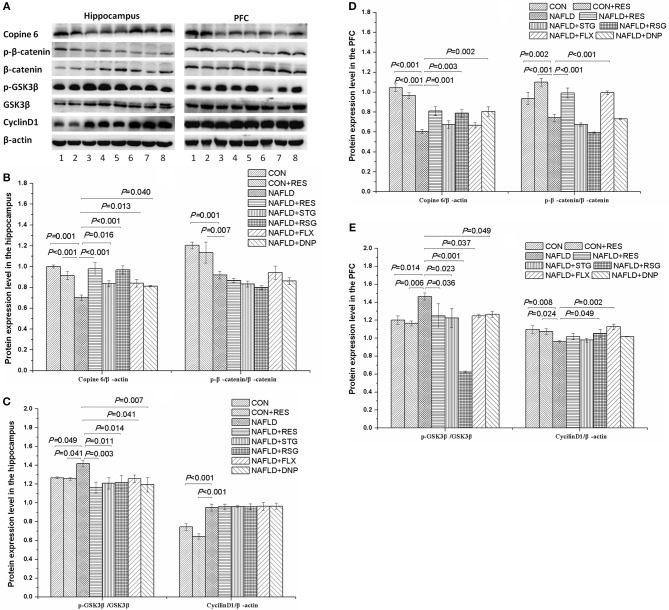
Effect of RES on the protein expression level of Copine 6, p-β-catenin, β-catenin, p-GSK3β, GSK3β, and cyclin D1 in the hippocampus and PFC of NAFLD rats. **(A)** shows a typical graph, and **(B–E)** show a statistical analysis of the Western blotting results. The data in panels **(B,C)** are presented as the mean ± SEM, with *n* = 3 for each group. The protein expression level of Copine 6 and p-catenin/catenin was decreased, whereas the protein expression level of p-GSK3β/GSK3β was increased in the hippocampus and PFC of NAFLD rats. The expression level of cyclin D1 was increased in the hippocampus but decreased in the PFC of NAFLD rats. Treatment with RES restored the imbalanced expression level of Copine 6 and p-GSK3β/GSK3β to normal in both the hippocampus and PFC and normalized the expression level of p-catenin/catenin in the hippocampus, without any notable effect on that of cyclin D1. 1 control; 2 CON + RES; 3 NAFLD; 4 NAFLD + DNP; 5 NAFLD + STG; 6 NAFLD + RSG; 7 NAFLD + FLX; 8 NAFLD + RES.

Figure 7 shows the protein expression levels of Copine 6, p-catenin, catenin, cyclin D1, p-GSK3β, and GSK3β in the hypothalamus and PFC of the rats. The protein expression levels of Copine 6 and p-catenin/catenin was decreased (*P* < 0.05 or *P* < 0.01), whereas the protein expression level of p-GSK3β/GSK3β was increased (*P* < 0.05 or *P* < 0.01) in the hippocampus and PFC of NAFLD rats. The expression level of cyclin D1 was increased in the hippocampus but decreased in the PFC of NAFLD rats (*P* < 0.05 or *P* < 0.01). Treatment of RES reversed the imbalanced expression levels of Copine 6 and p-GSK3β/GSK3β in both the hippocampus and PFC as well as that of p-catenin/catenin in the hippocampus (*P* < 0.05 or *P* < 0.01), without any notable effect on the expression level of cyclin D1.

## Discussion

**Figure 8:**
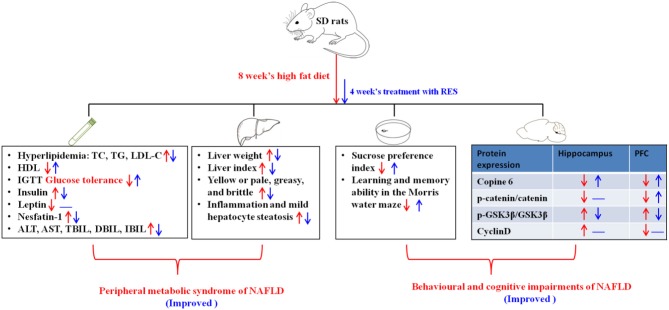
Summary of the therapeutic effect of RES on the peripheral metabolic syndrome and behavioral and cognitive impairments associated with NAFLD in a rat model. The red description and arrows indicate the dysfunction induced by 8 weeks on a high-fat diet, and the blue ones show the effects of four consecutive weeks of treatment with RES. The up arrows indicate increase, the down arrows indicate decrease, and the symbol “—” indicates that there is no significant change.

In the present study, our results showed that RES (15 mg/kg) administration could exert a therapeutic effect against NAFLD in rats, including not only metabolic and liver dysfunction but also behavioral and cognitive impairments such as anhedonia and decreased learning and memory ability in the MWM task. Moreover, the results showed that the effect of RES was partly attributable to its ability to regulate the abundance of nesfatin-1, which was significantly correlated with plasma lipid concentrations and behavioral performance. Furthermore, RES could also reverse the imbalanced protein expression levels of Copine 6 and the Wnt/β-catenin signaling pathway in the hippocampus and PFC of NAFLD rats. In Figure 8, we summarize the therapeutic effect of RES on the peripheral metabolic syndrome and behavioral and cognitive impairments of NAFLD in a rat model.

As a consequence of the pandemic spread of obesity, NAFLD is one of the leading causes of liver disease worldwide (3, 33). Although weight loss based on diet and exercise is the cornerstone treatment for NAFLD (4, 9) and percent weight reduction has been demonstrated to be correlated significantly with improvement in liver chemistry and histological activity (34), it is difficult to achieve and maintain. In the present study, high-fat diet-induced NAFLD rats showed obesity, but the drugs did not significantly decrease the gain of bodyweight.

With the remarkable evolution in the understanding of the pathogenesis of NAFLD, new medical therapies and even modifications of currently available therapies have gradually appeared (35, 36). Based on increasing experimental and clinical data, the efficacy of agents targeting insulin resistance has been demonstrated (37–39). Accordingly, STG and RSG, which are representative of DPP4 inhibitors and PPARγ agonists, were used as positive control drugs in the present study. Unsurprisingly, the results showed that treatment with STG or RSG could reverse hyperglycemia and hyperinsulinemia in NAFLD rats. In line with the effect of STG and RSG, RES could also restore the increased plasma glucose and insulin concentrations of NAFLD rats to normal, indicating its therapeutic effect on the dysfunction of glucose metabolism in NAFLD.

The development of NAFLD comes from an imbalance between the influx and production of fatty acids, and dyslipidemia and insulin resistance are reciprocal causes underlying the development of NAFLD. In the vicious cycle of insulin resistance and increased lipolysis, excess lipids eventually accumulate in lipid droplets, creating a fatty and inflamed liver (35). Consistently, in this study, the NAFLD rats presented increased liver mass and index and elevated plasma concentrations of TC, TG, LDL-C, TBIL, and liver enzymes, together with inflammation and mild hepatocyte steatosis in the liver. It has been reported that STG and RSG could suppress lipid accumulation and improve insulin resistance (15, 40). Consistently, in our present study, treatment with STG and RSG significantly improved the lipid accumulation in the blood and liver of NAFLD rats. Similarly, treatment with RES also reversed the observed hepatomegaly, dyslipidemia, hyperbilirubinemia, and hepatocyte steatosis of NAFLD rats. These results suggest a hepatoprotective and antihyperlipidemic effect of RES on NAFLD rats. FLX and DNP are other two positive control drugs used in this study to observe the behavioral performance of rats. However, lipid metabolism abnormalities have been reported in patients with depression treated with FLX (41) and patients with AD treated with DNP (42), and increased incidence of NAFLD has been found in male rat offspring exposed to FLX during the fetal and neonatal periods (43). In the present study, they showed no effect on the increased plasma glucose and lipid concentrations and the morphological and functional impairments in the liver of NAFLD rats. Additionally, we cannot absolutely exclude the synergistic effect of FLX or DNP and the high-fat diet on the lipid metabolism abnormalities and liver injuries of NAFLD rats. Interestingly, the plasma HDL-C concentration of NAFLD rats was increased after four consecutive weeks on a high-fat diet in our previous study (8) but decreased in the present study after eight consecutive weeks of high-fat diet feeding, which might suggest a compensatory mechanism and dynamic changes in HDL-C during the progression of NAFLD. Additionally, treatment with RES could increase the plasma HDL-C concentration in NAFLD rats. Together with the results of other studies using a hyperuricemia-related NAFLD rat model (44), these results indicated the therapeutic effect of RES on the NAFLD state.

Nesfatin-1 is an anorexic factor regulating feeding and metabolism. Consistent with our previous study (8), the plasma concentration of nesfatin-1 was also increased in the NAFLD rats, and significant correlations with the plasma levels of lipids, insulin, and leptin were observed. Although treatment with RES decreased the plasma concentration of nesfatin-1 in NAFLD rats, it had no significant effect on the plasma leptin level. Moreover, treatment with STG or RSG did not lower the plasma nesfatin-1 level. These results indicated an important role of nesfatin-1 in abnormal glucose and lipid metabolism in NAFLD rats and suggested a potential mechanism through which RES might exert its therapeutic effect on NAFLD, partially distinct from the mechanisms of STG and RSG.

Increasing evidence has demonstrated the role of nesfatin-1 in regulating neuropsychiatrically relevant behavior, including mood and cognition (26, 45), and plasma nesfatin-1 levels have been reported to be positively correlated with the severity of depression (46). In line with our previous study (8), the NAFLD rats in the present study showed a nesfatin-1-related decline in their SPI in the SPT, together with an impairment of learning and memory ability in the MWM task. However, there was no significant difference among groups with regard to the behavioral performance of rats in the OFT and TST, indicating that all the rats in this study were at the similar level of locomotion and exploration, anxiety, and despair behaviors. In the SPT, FLX-treated NAFLD rats showed an increased SPI, which was consistent with the clinical use of FLX in the treatment of depression. Similarly, DNP-treated NAFLD rats showed an improvement of the learning and memory in the MWM task. In line with findings from animal and human studies that STG produced cognitive improvement and antidepressant effects (16, 17), our results showed that STG treatment could improve the learning and memory of NAFLD rats and increase their SPI, which is taken as a measure of anhedonia. Importantly, these abnormal neurobehavioral changes could also be reversed by treatment with RES. Together with the negative correlation between plasma nesfatin-1 concentration and anhedonic behavior, the results confirmed the important role of nesfatin-1 in the pathogenesis of NAFLD-induced neurobehavioral impairments in rats. However, only RES and FLX, rather than STG or RSG, decreased the plasma nesfatin-1 concentrations of NAFLD rats. Considering the different effects of the positive control agents used in the present study, these results indicated that there might be some different mechanisms underlying the therapeutic effect of RES and other agents targeting insulin resistance.

The hippocampus and PFC are crucial brain areas involved in functions such as cognition and mood regulation. It has been reported that the Wnt/β-catenin signaling pathway plays important roles in the structure and function of the adult hippocampus and PFC, and impairment of Wnt/β-catenin signaling is involved in the pathogenesis of depression and AD (47, 48). Abnormally active GSK3β has been demonstrated to increase the susceptibility to depression-like behavior (49), memory impairment (49, 50), and impaired hippocampal neural precursor proliferation (49). In line with our previous findings (8), the present study found that the protein expression level of p-β-catenin/β-catenin was decreased, whereas the protein level of p-GSK3β/GSK3β was increased in the hippocampus and PFC of NAFLD rats. The expression level of cyclin D1 was increased in the hippocampus but decreased in the PFC of NAFLD rats. Treatment with RES could restore the imbalanced expression level of p-GSK3β/GSK3β to normal in both the hippocampus and the PFC and restore normal expression levels of p-β-catenin/β-catenin in the hippocampus, without any notable effects on the expression level of cyclin D1. Together with the effect of RES on the activity of the Wnt/β-catenin pathway in other studies (51, 52), our results suggest that the Wnt/β-catenin pathway underlies the neuroprotective mechanism of RES.

The calcium sensor Copine 6 has also been reported to play an important role in regulating neurotransmission, synaptic plasticity, and learning and memory (27, 28, 53). It has been demonstrated that Copine 6 is recruited from the cytosol of dendrites to postsynaptic spine membranes by calcium transients, linking the calcium signals to spine structural plasticity necessary for learning and memory (27). Moreover, a decreased protein expression level of Copine 6 has been demonstrated in the hippocampus and PFC of NAFLD (8) or stressed rats (29). In the present study, the results showed that treatment with RES increased the expression level of Copine 6 in the hippocampus and the PFC of NAFLD rats. Consider the finding that RES could induce the release of Ca^+2^ in a time-dependent manner (52), it might be rational to suppose that Ca^+2^ might play an important role underlying the mechanism of RES, which should be investigated in detail in the future studies.

In conclusion, the results of the present study demonstrated that RES could exert a therapeutic effect on a NAFLD rat model, not only on the dysfunction of liver and glucolipid metabolism but also on behavioral and cognitive impairments. Furthermore, increased plasma nesfatin-1 concentrations might be a link between the dysfunction of glucolipid metabolism and the behavioral and cognitive impairments observed in NAFLD rats, relating significantly to both plasma lipid concentrations and behavioral performance. Additionally, apart from its ability to decrease nesfatin-1 abundance, the therapeutic effect of RES on NAFLD rats might also be related to an imbalance in the expression level of Copine 6 and key proteins in the Wnt/β-catenin signaling pathway in the hippocampus and PFC.

## Data Availability

The datasets generated for this study are available on request to the corresponding author.

## Ethics Statement

All experimental procedures in the present study were approved by the Animal Care and Use Committee of Anhui Medical University, in compliance with the National Institutes of Health Guide for the Care and Use of Laboratory Animals (NIH publication No. 85-23, revised 1985).

## Author Contributions

J-FG designed the experiment. X-XC and Y-YX carried out the experiment and wrote the manuscript with support from J-FG. RW and ZC contributed to the animal experiment. KF and Y-XH performed the behavioral tests. YY and L-LH helped with the preparation. LP and J-FG supervised the project. All authors helped shape the analysis, research, and manuscript.

### Conflict of Interest Statement

The authors declare that the research was conducted in the absence of any commercial or financial relationships that could be construed as a potential conflict of interest.
